# Supplementary material for: Patterns of Intron Gain and Loss in Fungi
Source: PLoS Biol. 2004 Nov 30;2(12):e422. doi: 10.1371/journal.pbio.0020422 (PMC532390; doi:10.1371/journal.pbio.0020422)
Supplement: Table S1 — Also available at http://genes.mit.edu/NielsenEtAl/. (4.3 MB ZIP). [file pbio.0020422.st001.zip › NielsenEtAl/html/1205.html]

AN2314.1.NCU05429.1.MG03186.1.FG04313.1


```
 CLUSTAL W (1.82) Multiple Sequence Alignments - Introns Inserted


Sequence 1: MG03186.1	691 aa
Sequence 2: FG04313.1	707 aa
Sequence 3: NCU05429.1	733 aa
Sequence 4: AN2314.1	686 aa
Alignment Length: 736 aa
Number Identitical Residues: 426 aa
Alignment Score (without introns) 20871


MG03186.1 	---MATNGSNTVVANEG-------TVEHI--~--G1AVKLDPWLEPFEDVIKRRHGMAED
NCU05429.1	MNDISDNSSNSL-ANGGTVANSDIPKDGTGI2CAG~VLSIDPWLEPFKDTLKSRYSKAQS
FG04313.1 	MASIVKNSSNSLDPNQANGAAGDIPNDGT--~--G1VVKLDPWLEPFSGALKRRYSKAQD
AN2314.1  	---MTSTA----------------PSDGT--~--G1IIDLDPWLEPFREAIKRRFDYVES
          	   :  ..                . :       *  :.:*******  .:* *.. .:.

MG03186.1 	WLKKIEQSEGSIEKFTR0GTEVYGFNVKPDNTIVYREWAPNATAAFLVGDFN1QWSRQSH
NCU05429.1	WIDTINKTEGGLEKFSR0GTEIFGFNVDKDNTITYREWAPNAKQASLIGEFN1NWDRNAH
FG04313.1 	WIKTINAAEGGLEKFSR0GAEKFGFNVDANNNIVYREWAPNATAAYLIGDFN1GWNRGAH
AN2314.1  	WIKTVDEVEGGLDKFSK0GYEKFGFNVSETGDITYREWAPNAIEAALVGDFN1NWDTKAN
          	*:..::  **.::**:: * * :****.  . *.********  * *:*:**  *.  ::

MG03186.1 	PMKKNEYGVFEITVPPTAAGEPAIPHNSKVK0ISLFLPNGQLIDRLPAWIKYVTQDLSVS
NCU05429.1	RMKKNEFGVFEITIPPTANGQPAIPHNSKIK0ITLELPDGQWVDRLPAWIKYVTQDLSVS
FG04313.1 	PMKKNDFGVFEITLP-AQNGQAAIPHNSKLK0ISLDLPSGEHVDRLPAWIKYVTQDLSVS
AN2314.1  	PMTRDNFGVWEIALP-AKNGTPVIPHDSKVK0ITMVTRSGERIYRIPAWIKRVVQDLNVS
          	 *.::::**:**::* :  * ..***:**:* *::   .*: : *:***** *.***.**

MG03186.1 	PAYDARFWNPPSSERYTFKNPRPKKPKSVRVYEAHVGISSPELRVATYKEFTKNMLPRIK
NCU05429.1	PAYEARFWNPPQSERYTFKHKRPSKPESLRIYEAHVGISSPECKVATYKEFTKTMLPRIK
FG04313.1 	PAYDARFWNPPASETYKFKNSRPKKPASARVYEAHVGISSPEQKVASYKEFTKNMLPRIK
AN2314.1  	PIYESVFWNPPKAERYNFQHARPKKPESLRIYEAHVGISSPDTRVATYKEFTANMLPRIK
          	* *:: ***** :* *.*:: **.** * *:**********: :**:***** .******

MG03186.1 	KLGYNVIQLMAIMEHAYYASFGYQINNFFAASSRYGTPEDLKELIDAAHGMGLTVLLDVV
NCU05429.1	NLGYNAIQLMAIMEHAYYASFGYQVNSFFAASSRYGPPEDLKELVDTAHAMGIAVLLDVV
FG04313.1 	ALGYNVIQLMAVMEHAYYASFGYQINNFFAASSRYGTPEELKELIDTAHGLGITMLLDVV
AN2314.1  	YLGYNAIQLMAIMEHAYYASFGYQVNNFFAASSRYGKPEDLKELVDTAHSMGLVVLLDVV
          	 ****.*****:************:*.********* **:****:*:**.:*:.:*****

MG03186.1 	HSHASKNVLDGINEFDGTDHQYFHAGGKGRHDQWDSRLFNYGHHEVLRFLLSNLRFWMDE
NCU05429.1	HSHASKNVLDGLNEFDGTDHQYFHGGGRGKHDLWDSRLFNYGHHEVMRFLLSNLRFWMDE
FG04313.1 	HSHASKNVLDGINEFDGTDHQYFHGGGKGRHDQWDSRLFNYGHHEVMRFLLSNLRFWMDE
AN2314.1  	HSHASKNVDDGLNMFDGSDHLYFHSGSKGQHELWDSRLFNYGNHEVLRFLLSNLRFWMEE
          	******** **:* ***:** ***.*.:*:*: *********:***:***********:*

MG03186.1 	YQFDGFRFDGVTSMLYLHHGMG-~-T2GFSGGYHEYFGPDADEEALVYLMLANKMLHELY
NCU05429.1	YAFDGFRFDGVTSMLYLHHGIG-~-T2GFSGGYHEYFGPDVDEEAVVYLMLANEMLHQLY
FG04313.1 	YQFDGFRFDGVTSMLYVHHGMG-~-T~GFSGGYHEYFGSDVDEEAVVYMMLANEMLHQLY
AN2314.1  	YGFDGFRFDGVTSMLYTHHGIGT2VS~GFSGGYHEYFGPAVDDDGVMYLALANEMLHRLY
          	* ************** ***:*:  : ***********. .*::.::*: ***:***.**

MG03186.1 	PEVISIAEDVSGMPTLCLPWSDGGVGFDYRLAMAIPDMWIKILKEKKDEEWDMADICWTL
NCU05429.1	PNVITVAEDVSGMPALCLPLSLGGVGFDYRLAMAIPDMWIKILKEKKDEEWDMANITWTL
FG04313.1 	PEVITIAEDVSGMPALCVPLSLGGIGFDYRLAMAIPDMWIKILKEVKDDEWDIANICHTL
AN2314.1  	PDCITVAEDVSGMPALCLPHGLGGVGFDYRLAMAIPDMYIKLLKEKSDNDWDIGNLAFTL
          	*: *::********:**:* . **:*************:**:*** .*::**:.::  **

MG03186.1 	TNRRHGEKTIAYCESHDQA2LVGDKTLMMHLCDAEMYTHMSTLTPLTPIIDRGMALHKMI
NCU05429.1	TNRRHGEKTIAYCESHDQA2LVGDKTLMMHLCDAELYTNMSILTPLTPVIDRGMALHKMI
FG04313.1 	TNRRHGEKTIAYAESHDQA2LVGDKTLMMHLCDAEMYTHMSTLSPLTPVIDRGMALHKMI
AN2314.1  	TNRRHGEKTIAYAESHDQA2LVGDKSLMMWLCDKEMYTHMSVLTEFTPVIERGMALHKMI
          	************.****** *****:*** *** *:**:** *: :**:*:*********

MG03186.1 	RLLTHGLGGEGYLNFEGNEFGHPEWLDFPREGNQNSFWYARRQLNLTEDNLLRYRFLNDF
NCU05429.1	RLLTHSLGGEGYLNFEGNEFGHPEWLDFPREGNQNSFWYARRQLNLTEDGLLRYQYLNNF
FG04313.1 	RLVTHGLGGEGYLNFEGNEFGHPEWLDFPREGNNNSFWYARRQLNLTDDPLLRYKFLDNF
AN2314.1  	RLVTHALGGEGYLNFEGNEFGHPEWLDFPRAGNNNSFWYARRQLNLTEDHLLRYRFLNEF
          	**:**.************************ **:*************:* ****::*::*

MG03186.1 	DAAMNNCEEKHGWLSAPQAYISLKNESDMVVVFERAGVVFAFNFHPTQSFADYRIGVDVS
NCU05429.1	DRSMNLTEDKYGWLHAPQAYISLKHEGDKVIVFERAGLVFVFNFHPSNSYTDYRIGIEQA
FG04313.1 	DRMMNQTEAKYGWLSAPQAYISLKHEGDKVIVFERAGLVFIFNFHPTNSFSDYRIGIEVP
AN2314.1  	DRAMQLTESKYGWLHAPQAYISLKHEGDKVLVFERADLLWIFNFHPTESFTDYRVGVEQA
          	*  *:  * *:*** *********:*.* *:*****.::: *****::*::***:*:: .

MG03186.1 	GTYKVALTTDDKEFGGHGRIDSSTRFFTTPMEWNGRKNWTHIYLPSRTAV0VL~ELE---
NCU05429.1	GTYRIVLDSDTKEHGGFNRLDPQTRFFTSDLPWNNRKNSTHVYIPARTAF~VS1PCARVH
FG04313.1 	GTYRVVLNSDHGDVGGHNRIDENTRFFTTPMEWNNRKNWTHVYIPSRTAI~IL~ALESTV
AN2314.1  	GTYRVVLDTDDQAFGGLGRIDQGTRFFTTDMEWNGRRNYLQVYIPTRTAL0AL~ALEETL
          	***::.* :*    ** .*:*  *****: : **.*:*  ::*:*:***.          

MG03186.1 	--------------------------
NCU05429.1	PFRVGYERPDIPSVVQVRVMRITAAG
FG04313.1 	TQSS----------------------
AN2314.1  	--------------------------
          	
```
